# ASSOCIATION BETWEEN MULTIPLE FRAILTY INDICES AND CVD MORTALITY

**DOI:** 10.1093/geroni/igad104.1653

**Published:** 2023-12-21

**Authors:** Benjamin Seligman, Saadia Qazi, Manas Rane, Sarah Preis, Ariela Orkaby, J Michael Gaziano, Jane Driver

**Affiliations:** UCLA School of Medicine, Los Angeles, California, United States; Brigham and Women’s Hospital, Boston, Massachusetts, United States; West Roxbury VA Medical Center, West Roxbury, Massachusetts, United States; Boston University School of Public Health, Boston, Massachusetts, United States; Harvard Medical School, Boston, Massachusetts, United States; Brigham and Women’s Hospital / VA Boston, Boston, Massachusetts, United States; VA Boston Heralthcare System, Boston, Massachusetts, United States

## Abstract

Introduction Frailty, a syndrome of physiologic vulnerability, increases cardiovascular disease (CVD) risk. Which frailty tool is ideal for risk stratification remains unclear. We calculated three frailty scores from the Million Veteran Program (MVP) and examined their association with mortality and CVD in older Veterans. Methods Participants were from MVP - a large, contemporary Veteran cohort study - and aged ≥50 years at baseline (2011-2018). The frailty scores used were: MVP-FI (36-item questionnaire deficit accumulation frailty index), VA-FI (31-item EHR index), and modified Study of Osteoporotic Fractures (mSOF; a physical frailty score). MVP-FI and VA-FI scores of ≤0.10 were robust, 0.11-0.20 pre-frail, and ≥0.21 frail; mSOF scores of 0 were robust, 1 pre-frail and ≥2 frail. Primary outcomes were all-cause and CVD mortality. Secondary outcomes were incident stroke, myocardial infarction (MI), and heart failure (HF). Cox regression was used to evaluate the association of frailty with outcomes. Results Among 190,688 participants, mean age was 69 ±9, 94% were male. By MVP-FI, 29% were robust, 42% pre-frail, and 29% frail. Hazard ratios (HR, 95% CI) for all-cause mortality were 1.66 (1.61-1.72) and 3.05 (2.95-3.16) for pre-frailty and frailty, respectively. For CVD mortality, HRs were 1.76 (1.65-1.88) and 3.65 (3.43-3.90) for pre-frailty and frailty. Hazards of stroke, MI, and HF also increased with greater frailty. VA-FI and mSOF yielded concordant results. Conclusion Irrespective of measure, frailty is associated with increased all-cause mortality and CVD event risk. Clinicians and researchers may consider the most convenient tool for available data to incorporate frailty into practice.

